# Prevalence of Coronary Artery Diseases Among Adult Women Residents of Tehran: A Cross‐Sectional Report From Tehran Cohort Study (TeCS)

**DOI:** 10.1155/crp/1614045

**Published:** 2026-08-06

**Authors:** Amirhossein Heidari, Nazila Heidari, Arash Jalali, Yekta Ghane, Zahra Lotfi, Akbar Shafiee, Farshid Alaeddini, Hamidreza Raeisi-Dehkordi, Saeed Sadeghian, Mohammadali Boroumand, Abbasali Karimi, Oscar H. Franco

**Affiliations:** ^1^ Cardiac Primary Prevention Research Center, Cardiovascular Diseases Research Institute, Tehran University of Medical Sciences, Tehran, Iran, tums.ac.ir; ^2^ Faculty of Medicine, Tehran Medical Sciences, Islamic Azad University, Tehran, Iran, azad.ac.ir; ^3^ School of Medicine, Iran University of Medical Sciences, Tehran, Iran, iums.ac.ir; ^4^ Tehran Heart Center, Cardiovascular Diseases Research Institute, Tehran University of Medical Sciences, Tehran, Iran, tums.ac.ir; ^5^ Department of Epidemiology and Biostatistics, School of Public Health, Tehran University of Medical Sciences, Tehran, Iran, tums.ac.ir; ^6^ School of Medicine, Tehran University of Medical Sciences, Tehran, Iran, tums.ac.ir; ^7^ Department of Global Public Health and Bioethics, Julius Center for Health Sciences and Primary Care, University Medical Center (UMC) Utrecht, Utrecht University, Utrecht, the Netherlands, uu.nl

**Keywords:** CAD, coronary artery disease, prevalence, Tehran, Tehran Cohort Study, women

## Abstract

**Background:**

Coronary artery disease (CAD) is the leading cause of death worldwide. Among women, CAD is often underestimated, resulting in underdiagnosis and undertreatment. This study aimed to assess the prevalence of CAD and its risk factors in adult women, using data from the Tehran Cohort Study (TeCS).

**Methods:**

We analyzed data from 4478 women with complete CAD information collected during the TeCS recruitment phase. The age‐weighted prevalence of CAD was estimated using the 2016 national census data and previous CAD diagnoses. Logistic regression models were applied to identify factors independently associated with CAD in women.

**Results:**

The overall prevalence of CAD was 4.48% (mean age: 53.0 ± 12.37 years), with an age‐weighted prevalence of 4.1% (95% CI: 3.1%–5.4%). Increasing age was strongly associated with CAD, with women aged ≥ 75 years showing markedly higher odds (OR: 11.94, 95% CI: 3.93–36.31, *p* < 0.001). Hypertension (OR: 2.58, 95% CI: 1.75–3.80, *p* < 0.001), hyperlipidemia (OR: 2.25, 95% CI: 1.55–3.24, *p* < 0.001), and diabetes mellitus (OR: 2.07, 95% CI: 1.49–2.88, *p* < 0.001) were also significant determinants of CAD. Older age groups exhibited a higher prevalence of CAD risk factors. Within the CAD group, a greater proportion of women had multiple comorbid risk factors compared with their non‐CAD counterparts in the same age categories.

**Conclusion:**

The prevalence of CAD and its associated risk factors among women in Tehran is substantial. These findings highlight the need for targeted preventive strategies to reduce CAD incidence in vulnerable female populations.

**Highlights**
• Coronary artery disease (CAD) in women remains underdiagnosed and undertreated, highlighting the need for focused research and tailored interventions.
• Among women aged ≥ 35 years in Tehran, the age‐weighted prevalence of CAD was 4.1%, with strong associations to hypertension, hyperlipidemia, diabetes mellitus, and older age.
• Women with CAD, particularly in older age groups, demonstrated a higher burden of multiple comorbid risk factors compared with their non‐CAD counterparts.
• These findings emphasize the urgency of implementing targeted preventive strategies to mitigate CAD risk in vulnerable female populations.

## 1. Introduction

Cardiovascular diseases (CVDs) are the leading global cause of death, responsible for approximately 18.6 million deaths worldwide in 2019 [1]. Additionally, CVDs accounted for 393.1 million disability‐adjusted life years (DALYs). Coronary artery disease (CAD) is the most prevalent non‐communicable CVD in low‐ and middle‐income countries, causing around seven million deaths and 129 million DALYs annually, thus imposing a significant economic burden globally [2].

Similarly, CVDs are one of the leading causes of mortality in Iran [3]. In 2019, Iran was among the countries with the highest age‐standardized prevalence rates of CVD. The CVD burden in Iran is projected to more than double the levels recorded in 2005 by 2025 [4]. CAD‐related deaths constitute approximately 39.3% of annual total mortality in Iran [5]. The Global Burden of Disease (GBD) report from 2015 identified Iran as one of the countries with the highest prevalence of CVD, with an incidence rate of 9000 CVD cases per 100,000 individuals [4]. Moreover, the prevalence of CAD and its risk factors is higher in Iran and Middle Eastern countries compared to Western countries [6]. Significant progress has been made in identifying and managing modifiable risk factors for CAD within community settings over the past 6 decades [7]. Despite these advancements, CAD remains the primary cause of mortality, morbidity, and disability worldwide [8].

CVDs, including CAD, are the predominant cause of mortality among women in the United States, accounting for one‐third of all female fatalities [9]. Furthermore, 45% of women over 20 years of age suffer from some form of CVD [10]. However, substantial differences exist between men and women in the manifestations, diagnosis, management, and outcomes of CVDs, resulting in higher rates of rehospitalizations and mortality in women compared to men [11]. Known CVD risk factors, such as diabetes mellitus (DM) and smoking, have a greater impact on women than on men. Additionally, women have specific risk factors for CVD, such as polycystic ovary syndrome and pregnancy‐associated conditions, which contribute to an increased risk of CVD in the future [10, 12].

Regarding the development of CAD, both men and women are at increased risk after the age of 40 [13]. However, women’s coronary arteries undergo significant changes seven to 10 years later than those of men [14]. Compared to men, women tend to present with atypical anginal symptoms more frequently and exhibit lower sensitivity to noninvasive diagnostic tests for CAD. Additionally, women are less likely to undergo coronary angiography and revascularization.

The issue of CAD is underestimated in women, leading to its underdiagnosis and undertreatment. The most effective way to optimize women’s cardiovascular health is to pay particular attention to sex‐specific factors in their care. While sex‐specific CVD data have been steadily increasing, it is rarely collected or used in clinical practice. Although previous research has focused on CVDs in the general population of Iran [2, 4, 15], the substantial prevalence of CAD among women necessitates a comprehensive investigation to provide up‐to‐date data on the epidemiology of CAD for future health strategies. We herewith aimed to evaluate the prevalence of CAD and its associated risk factors among women aged 35 years and older, using data from a large sample of participants in the Tehran Cohort Study (TeCS).

## 2. Methods

### 2.1. Study Design and Participants

This cross‐sectional study utilized data from the recruitment phase of the TeCS, an ongoing population‐based study in Tehran, Iran, conducted between March 2016 and March 2019. The study protocol and methodology have been previously published [16]. Briefly, a random sampling method was used to include households with adults aged 35 years and older from all 22 districts of Tehran. A total of 4215 households, comprising 8296 adults aged 35 years or older, participated in TeCS during this period. Demographic characteristics, education level, metabolic risk factors, physical activity status, pre‐existing comorbidities, past medical history, drug history, and family history (defined PFHxCAD as the occurrence of CAD before the age of 55 in male relatives and 65 in female relatives, respectively) were collected through face‐to‐face interviews and a predesigned checklist. Additionally, anthropometric and physiological indices, as well as biochemical and laboratory tests, were measured for each participant. Female participants with complete CAD data were eligible for inclusion. Ultimately, data from 4478 individuals were selected for statistical analyses (Figure 1). The TeCS protocol was approved by the Deputy of Research and the Ethics Committee of Tehran University of Medical Sciences (IR.TUMS.MEDICINE.REC.1399.074). All participants received information about the survey before enrollment, and written consent was obtained from those who agreed to participate.

**FIGURE FIGURE1:**
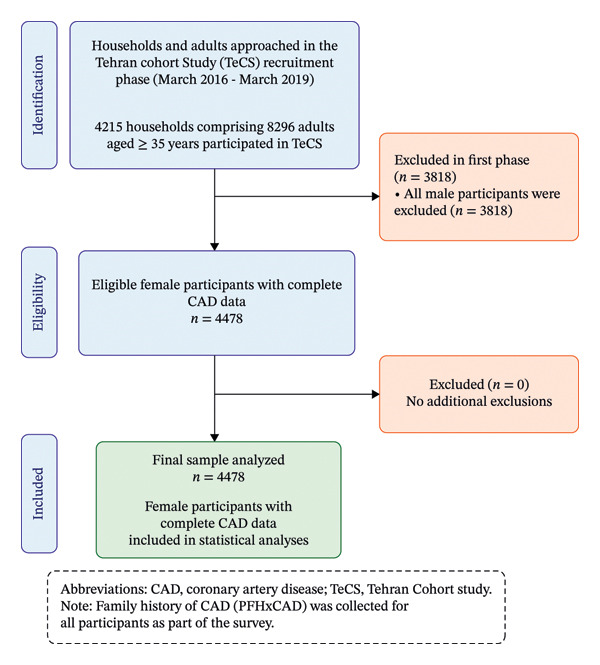
Flowchart of participant selection in the Tehran Cohort Study (TeCS), including recruitment of adults aged ≥ 35 years and final inclusion of 4478 eligible female participants with complete coronary artery disease (CAD) data for statistical analyses.

### 2.2. Data Collection

In this study, women aged ≥ 35 years were evaluated for sociodemographic data (i.e., ethnicity, occupation, education, and marital status), and cardiovascular characteristics such as family history of CVD, previous history of CAD and/or cerebrovascular disease, prior history of cardiac intervention (i.e., percutaneous coronary intervention [PCI] and cardiac artery bypass grafting), hypertension, hyperlipidemia, DM, and tobacco and alcohol consumption. In addition, lifestyle, habits, and health behaviors, including physical activity level, medical history, anthropometric variables, and biochemical and laboratory tests, were assessed. Anthropometric indices, including height, weight, waist circumference, and hip circumference, were measured by an experienced nurse. Body mass index (BMI) was calculated as weight divided by the squared value of height (kg/m^2^). Hip circumference was measured around the broadest part of the buttocks, and waist circumference was measured at the midpoint between the inferior margin of the lowest palpable rib and the superior border of the iliac crest. Venous blood samples were obtained from individuals after 8–12 h of fasting and handled at field stations according to appropriate standards to measure levels of fasting blood sugar (FBS), creatinine, and lipid profiles, including triglycerides (TG), high‐density lipoprotein cholesterol (HDL), and low‐density lipoprotein cholesterol (LDL). Blood pressure (BP) was measured using a digital brachial cuff sphygmomanometer (M6 Comfort Omron, Omron Healthcare, Kyoto, Japan) with a suitable cuff size in a sitting position after at least 5 minutes of rest. Following recent guidelines, this measurement was performed at least twice within a four‐hour period.

### 2.3. Definition of Variables

Participants were classified as having CAD if they had a self‐reported history confirmed by documentation, including multidetector computed tomography coronary angiography, myocardial perfusion imaging, prior coronary artery bypass grafting (CABG) or PCI, invasive coronary angiography, or a documented history of myocardial infarction (MI). Additional cardiovascular characteristics assessed included systolic and diastolic BP, valvular heart disease, cerebrovascular disease, and family history of CVD, based on self‐report or available records. In women without CAD, the validated Persian version of the Rose Angina Questionnaire was administered to evaluate chest pain and ischemic symptoms [17]. Pre‐existing comorbidities were defined as follows: hypertension, hyperlipidemia, and DM were identified by a prior clinical diagnosis or the use of relevant medications. Chronic kidney disease (CKD) was defined as a documented history of kidney failure, abnormal test results, regular use of kidney‐related medication, dialysis, or prior kidney transplantation. The history of abortion was defined as any type of abortion at any stage of pregnancy. Oral contraceptive pill (OCP) use refers to the consumption of any OCP at any point in a woman’s life.

Current tobacco smoking was defined as regular or occasional use of cigarettes, hookah, pipe, or water pipe at the time of the interview. Participants who reported no current smoking but had a past history of smoking, or who had quit at least 1 month before the interview, were classified as former smokers. Alcohol use was defined as the consumption of any alcoholic beverage during the preceding 12 months. Opium use was defined as any past or present use of opium or its derivatives, either orally or by inhalation.

Physical activity was assessed using a subjective Likert scale during the in‐person interview and classified into three levels: low, intermediate, and high. CAD risk factors in this population were defined as DM, hypertension, dyslipidemia, cigarette smoking, and a positive family history of CAD. Based on these, participants were categorized into four groups: no risk factors, one risk factor, two risk factors, and three or more risk factors. Detailed definitions of these variables have been published in previous TeCS studies [18–21].

### 2.4. Statistical Analyses

Categorical variables were summarized as frequencies and percentages, while continuous variables were reported as means with standard deviations (SD) or medians with interquartile ranges (IQR), depending on their distribution. Normality was assessed using the Kolmogorov–Smirnov test. Group comparisons between CAD and non‐CAD participants were performed using the chi‐square test for categorical variables. For continuous variables, independent samples *t*‐tests were used when normally distributed and the Mann–Whitney U test when skewed. Age and laboratory parameters, including FBS, serum creatinine, LDL, HDL, and LDL/HDL ratio, were analyzed accordingly.

The age‐standardized prevalence of CAD in women was estimated based on the 2016 Iran Population and Housing Census, with 95% confidence intervals (CIs). Multivariable logistic regression models were applied to assess the association between baseline covariates and CAD, using clinically relevant variables selected based on previous TeCS studies [16, 21, 22]. The models were adjusted for age, education level, marital status, BMI, tobacco, opium, and alcohol use, physical activity level, abortion history, OCP use, diabetes, dyslipidemia, and hypertension. Adjusted associations were reported as odds ratios (ORs) with 95% CIs. All analyses were conducted using Stata software, version 16 (StataCorp LLC, College Station, TX, 2019).

## 3. Results

### 3.1. General Characteristics of the Study Population

Among 8296 participants, 4478 women with complete CAD information were included in the final analysis (mean age: 53.0 ± 12.37 years). The prevalence of CAD was 4.48%, with an age‐weighted prevalence of 4.1%. Women with CAD were significantly older than those without CAD (66.0 ± 9.5 vs. 53.0 ± 12.37 years, *p* < 0.001). CAD prevalence increased with age, reaching the highest rate (38.3%) among women aged 65–74 years.

CAD‐positive participants had a significantly higher mean BMI (30.2 vs. 28.6 kg/m^2^, *p* < 0.001), with a larger proportion classified as obese (BMI ≥ 30 kg/m^2^: 49% vs. 36.1%, *p* < 0.001). They also had greater abdominal circumference and waist‐to‐hip ratio (*p* < 0.001). A significantly higher proportion of CAD cases were housewives (*p* < 0.001) and had lower educational attainment, particularly higher rates of illiteracy (29.4%) and incomplete schooling (6–11 years: 41.3%) compared with the non‐CAD group (*p* < 0.001). Angina pectoris, assessed using the Rose questionnaire, was also more frequent among CAD cases (*p* < 0.001).

Comorbidities were more prevalent in women with CAD, including cerebrovascular disease (*p* < 0.001), history of abortion (*p* = 0.002), DM (*p* < 0.001), hyperlipidemia (*p* < 0.001), hypertension (*p* < 0.001), and elevated systolic BP (*p* < 0.001). Physical activity levels were significantly lower among the CAD group, with 43.4% reporting low activity compared to 18.3% in non‐CAD participants (*p* < 0.001).

Laboratory findings revealed significantly higher levels of serum creatinine, TG, LDL, and FBS, along with lower HDL levels in CAD cases (all *p* < 0.001). No significant differences were observed between groups in tobacco smoking, opium use, alcohol consumption, or OCP use.

All comparisons of demographic, anthropometric, and clinical characteristics between CAD and non‐CAD participants are presented in Table 1.

**TABLE 1:** Baseline characteristics of the study population according to coronary artery disease status in the Tehran Cohort Study (TeCS).

Demographic characteristics^∗^	Total population (*n* = 4478)	Non‐CAD group (*n* = 4266)	CAD group (*n* = 201)	*p* value ^#^
Age, mean ± SD, years	53.0 ± 12.37	52.4 ± 12.14	66 ± 9.5	< 0.001
Age subgroups, *n* (%)				< 0.001
35–44 years	1323 (29.5)	1314 (30.8)	4 (2)	
45–54 years	1202 (26.8)	1181 (27.7)	18 (9)	
55–64 years	1102 (24.6)	1040 (24.4)	60 (29.9)	
65–74 years	600 (13.4)	522 (12.2)	77 (38.3)	
≥ 75 years	251 (5.6)	209 (4.9)	42 (20.9)	
Ethnicity, *n* (%)				0.062
Fars	2182 (48.7)	2069 (48.5)	112 (55.7)	
Azari	1305 (29.1)	1248 (29.3)	57 (28.4)	
Other	981 (21.9)	948 (22.2)	32 (15.9)	
Occupation, *n* (%)				< 0.001
Employed	828 (18.5)	819 (19.2)	9 (4.5)	
Housewife	3197 (71.4)	3036 (71.2)	161 (80.1)	
Retired	370 (8.3)	341 (8)	28 (13.9)	
Unemployed	72 (1.6)	69 (1.6)	3 (1.5)	
Education, *n* (%)				< 0.001
Illiterate	427 (9.5)	368 (8.6)	59 (29.4)	
1–5 years	512 (11.4)	469 (11)	43 (21.4)	
6–11 years	2363 (52.8)	2280 (53.5)	83 (41.3)	
≥ 12 years	1165 (26.0)	1148 (26.9)	16 (8)	
Marital status, *n* (%)				0.971
Single	41 (0.9)	39 (0.9)	2 (1)	
Married	4283 (95.6)	4088 (95.8)	193 (96)	
Divorced	145 (3.2)	139 (3.3)	6 (3)	
ROSE angina pectoris, *n* (%)	346 (7.7)	296 (6.9)	50 (24.9)	< 0.001
Body mass index, mean ± SD (kg/m^2^)	28.7 ± 5.16	28.6 ± 5.13	30.2 ± 5.59	< 0.001
Body mass index subgroups, *n* (%)				< 0.001
< 25 kg/m^2^	1141 (25.5)	1110 (26.3)	31 (15.8)	
25–29.99 kg/m^2^	1661 (37.1)	1591 (37.6)	69 (35.2)	
≥ 30 kg/m^2^	1622 (36.2)	1526 (36.1)	96 (49)	
Abdominal circumference, *n* (%)	95.0 (12.38)	94.7 (12.33)	101.1 (11.82)	< 0.001
Waist–hip ratio, *n* (%)	0.890 (0.074)	0.9 (0.07)	0.9 (0.07)	< 0.001
History of abortion, *n* (%)	1473 (32.9)	1387 (32.5)	86 (42.8)	0.002
History of cerebrovascular disease, *n* (%)	55 (1.2)	46 (1)	9 (4.4)	< 0.001
OCP use, *n* (%)	1836 (41.0)	1743 (40.9)	93 (46.3)	0.128
Diabetes, *n* (%)	806 (18.0)	616 (14.4)	96 (47.8)	< 0.001
Hyperlipidemia, *n* (%)	1550 (34.6)	1401 (32.9)	149 (74.1)	< 0.001
Hypertension, *n* (%)	1321 (29.5)	1166 (27.3)	155 (77.1)	< 0.001
Family history of CVD, *n* (%)	504 (11.2)	486 (11.4)	18 (8.9)	0.286
Tobacco use, *n* (%)				
Current	346 (7.7)	3886 (91.1)	187 (93)	0.383
Former	46 (1.0)	43 (1)	3 (1.5)	
Never	4074 (91.0)	335 (7.9)	11 (5.5)	
Opium consumption, *n* (%)	24 (0.5)	22 (0.5)	2 (1)	0.293
Alcohol consumption, *n* (%)	139 (3.1)	137 (3.2)	2 (1)	0.078
Physical activity, *n* (%)				< 0.001
Low	863 (19.3)	777 (18.3)	86 (43.4)	
Intermediate	2688 (60.0)	2592 (61)	96 (48.5)	
High	896 (20.0)	879 (20.7)	16 (8.1)	
Chronic kidney diseases, *n* (%)	34 (0.8)	32 (0.8)	2 (1.0)	0.665
Systolic blood pressure, mean ± SD (mmHg)	119.2 ± 19.4	118.6 ± 19.1	131.9 ± 20.4	< 0.001
Diastolic blood pressure, mean ± SD (mmHg)	80.7 ± 10.7	80.7 ± 10.7	81.3 ± 11	0.438
Serum Creatinine, median (25th, 75th percentile) (mg/dL)	0.71 (0.64, 0.80)	0.71 (0.64, 0.8)	0.79 (0.7, 0.91)	< 0.001
Serum HDL, median [25th, 75th percentile] (mg/dL)	49.1 (12.64)	49.2 (12.68)	46.1 (11.6)	0.001
Serum LDL, median [25th, 75th percentile] (mg/dL)	112 [92, 137]	113 [92, 138]	92 [73.5, 117]	< 0.001
Serum Triglyceride, median [25th, 75th percentile] (mg/dL)	119 [84, 166]	118 [83, 165]	138.5 [105, 188]	< 0.001
Serum FBS, median [25th, 75th percentile] (mg/dL)	96 [89, 105]	95 [89, 104]	107.5 [97, 152]	< 0.001

Abbreviations: CAD, coronary artery disease; OCP, oral contraceptive pills.

^∗^Data are presented as mean ± standard deviation and median [interquartile range] for continuous variables and number (percentage calculated for rows) for categorical variables.

^#^ *p* < 0.05 was considered statistically significant.

### 3.2. Determinants Associated With CAD in Women

In the adjusted model, age was significantly associated with CAD, with older women showing markedly higher odds of disease (Table 2). Women aged 65–74 years (OR: 8.97, 95% CI: 3.09–26.05, *p* < 0.001) and ≥ 75 years (OR: 11.94, 95% CI: 3.93–36.31, *p* < 0.001) had the greatest risk compared with those aged 35–44 years. Although the 45–54 age group also demonstrated an increased risk (OR: 2.50, 95% CI: 0.83–7.55), this association was not statistically significant.

**TABLE 2:** Independent determinants associated with coronary heart disease among women in the Tehran Cohort Study (TeCS).

Characteristics	Odds ratio	95% confidence interval	*p* value^∗^
Age subgroups			< 0.001
35–44 years	Reference		
45–54 years	2.500	0.83–7.55	0.104
55–64 years	5.180	1.80–14.87	0.002
65–74 years	8.970	3.09–26.05	< 0.001
≥ 75 years	11.940	3.93–36.31	< 0.001
Education			0.022
Illiterate	Reference		
1–5 years	0.935	0.59–1.48	0.776
6–11 years	0.670	0.44–1.02	0.060
≥ 12 years	0.390	0.20–0.76	0.005
Marital status			0.143
Single	Reference		
Married	0.700	0.15–3.19	0.645
Divorced	1.730	0.30–10.16	0.541
Body mass index subgroups			0.999
< 25 kg/m^2^	Reference		
25–29.99 kg/m^2^	0.990	0.62–1.60	0.979
≥ 30 kg/m^2^	0.990	0.62–1.57	0.967
Tobacco use			0.948
Current	Reference		
Former	1.080	0.29–4.00	0.902
Never	0.890	0.42–1.91	0.772
Opium consumption	1.550	0.32–7.50	0.589
Alcohol consumption	0.700	0.14–3.50	0.666
Physical activity level			0.101
Low	Reference		
Intermediate	0.720	0.51–1.03	0.069
High	0.580	0.31–1.07	0.079
History of abortion	1.130	0.82–1.56	0.458
OCP use	1.120	0.82–1.56	0.495
Diabetes	2.070	1.49–2.88	< 0.001
Hyperlipidemia	2.250	1.55–3.24	< 0.001
Hypertension	2.580	1.75–3.80	< 0.001

Abbreviation: OCP, oral contraceptive pills.

^∗^ *p* < 0.05 was considered statistically significant.

Comorbidities were strongly linked to CAD, including DM (OR: 2.07, 95% CI: 1.49–2.88, *p* < 0.001), hyperlipidemia (OR: 2.25, 95% CI: 1.55–3.24, *p* < 0.001), and hypertension (OR: 2.58, 95% CI: 1.75–3.80, *p* < 0.001). By contrast, marital status, education, BMI, physical activity, use of opium, alcohol, or tobacco, as well as history of abortion and OCP use, were not significantly associated with CAD.

### 3.3. Comparison of the Number of Cardiovascular Disease Risk Factors and CAD

The distribution of risk factors varied across age groups (Figure 2). The proportion of women with no risk factors declined steadily with age, from 62.1% in the 35–44 age group to 13.5% among those aged ≥ 75 years. In contrast, the proportion of participants with two or more risk factors increased with advancing age.

**FIGURE FIGURE2:**
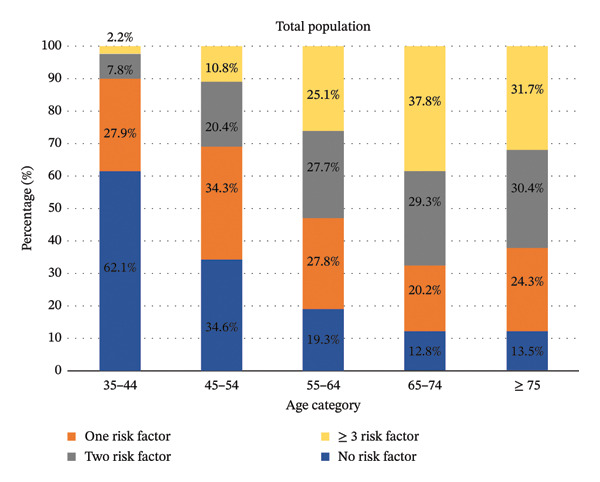
The prevalence of risk factors (diabetes, hypertension, dyslipidemia, smoking, and family history of coronary artery disease) across age categories within the total population.

**FIGURE 4:**
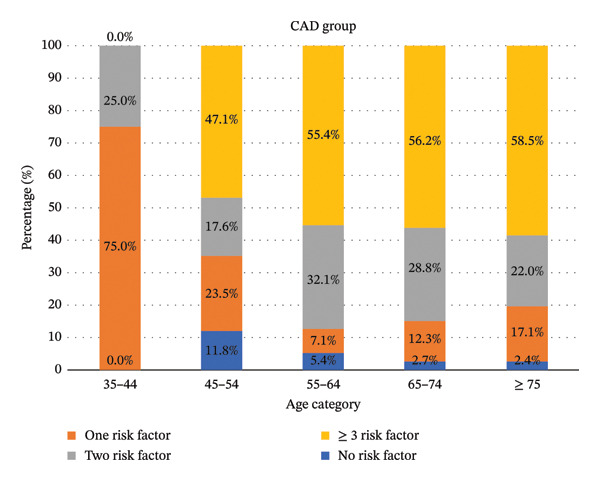
The prevalence of risk factors (diabetes, hypertension, dyslipidemia, smoking, and family history of coronary artery disease) across age categories within the CAD group.

In the non‐CAD group, the number of risk factors also showed an overall increasing trend with age (Figure 3). Notably, within each age category, women with CAD were more likely to have three or more risk factors compared with their non‐CAD counterparts (Figure 4). Conversely, the proportion of participants with no risk factors was consistently lower in the CAD group than in the non‐CAD group across all age categories.

**FIGURE FIGURE3:**
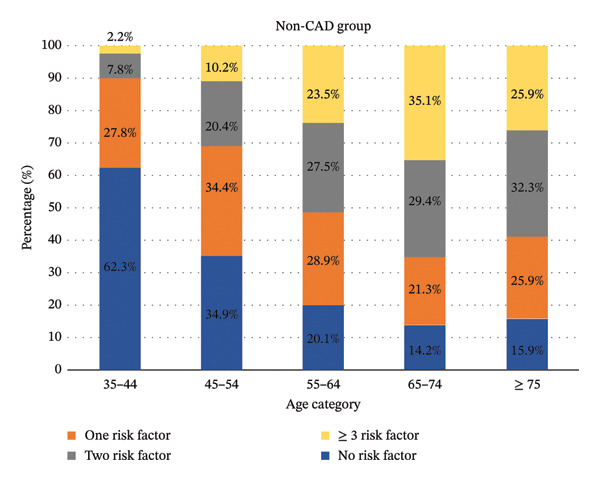
The prevalence of risk factors (diabetes, hypertension, dyslipidemia, smoking, and family history of coronary artery disease) across age categories within the non‐CAD group.

## 4. Discussion

This study evaluated the prevalence and risk factors of CAD among women aged ≥ 35 years in Tehran. As the first comprehensive investigation in this population, we found a CAD prevalence of 4.48%, with an age‐weighted prevalence of 4.1%. Older age, DM, hypertension, and hyperlipidemia were identified as independent determinants of CAD.

Tehran, the capital of Iran, is a densely populated urban center located in the north‐central region of the country [23]. According to the most recent national census, the greater Tehran metropolitan area is home to over 13 million individuals, representing a diverse range of socioeconomic and educational backgrounds [24]. The city comprises 22 administrative districts and is predominantly urban in structure, with minimal representation of rural or peri‐urban areas. Healthcare infrastructure in Tehran is comparatively well‐developed, offering wide access to preventive, diagnostic, and treatment services through both public and private sectors. Literacy rates exceed 90%, and the majority of residents have access to health insurance [25]. According to previous reports from the TeCS [16], the population exhibits a high prevalence of cardiometabolic risk factors, underscoring the importance of our study aim in this setting.

CAD is the leading single cause of morbidity, mortality, and loss of DALYs globally, increasing the risk of annual mortality in MI survivors five to sixfold compared to those without CAD [26]. Based on GBD data, CAD accounted for 16.2% of all‐cause mortality and 7.19% of DALYs globally in 2019 [27]. Although CAD mortality and prevalence vary across countries, it remains the most common cause of death across all income classes [26]. Notably, over 75% of CVD deaths occur in low‐ and middle‐income countries, indicating a significant impact on these nations [28]. One‐third of middle‐aged women and half of middle‐aged men in the USA are predicted to develop some symptoms of CAD [29]. In Western countries, CAD incidence rates have been reported at 200 to 500 per 100,000 person‐years for men and 60 to 150 per 100,000 person‐years for women [30]. Additionally, GBD reports that Iran had 593,000 new CAD cases in 2019, accounting for 26.2% of all deaths and 10.3% of all DALYs, exceeding the global average.

Various studies have yielded different results regarding CAD prevalence based on geographic location. According to a study conducted by Sarebanhassanabadi et al. in Yazd City, CAD incidence was reported in 14.5% of the total studied population, with incidence rates of 16.7% and 12.0% for males and females, respectively [27]. In another study in Borujerd City, definite CAD prevalence was reported in 17.1% of men and 19.9% of women in the total study population [31]. A report from Tehran stated that the age‐standardized incidence rate of CAD for males and females was 10.5 and 6.1 per 1000 person‐years, respectively [32]. Overall, CAD prevalence was reported in 4.8% of our study population, confirming that CAD prevalence differs among geographic locations.

In addition to geographical variation, CAD incidence is consistent with the distribution pattern of CAD risk factors. CAD risk factors can be categorized into non‐modifiable and modifiable risk factors. Non‐modifiable risk factors include age, sex, race, and family history of CAD, while high BP, abnormal blood lipid levels, DM, excess alcohol and opium consumption, cigarette smoking, obesity, psychosocial status, and sedentary behavior are classified as modifiable risk factors [33, 34]. Women are less likely to develop CAD than men of comparable age during their reproductive stage; however, this potential advantage diminishes after menopause [35]. Prior investigations have demonstrated that CAD tends to occur in females at a later age and with more comorbidities, particularly hypertension, DM, renal dysfunction, and previous congestive heart failure [36, 37]. Additionally, females are susceptible to women‐specific CAD risk factors, such as pregnancy‐related hypertension, gestational diabetes, miscarriage, and OCP consumption, which increase the incidence rate of CAD [38, 39]. In our study, although the history of abortion and OCP use was not significantly associated with CAD, they were more prevalent in CAD cases.

Advancing age is strongly associated with an increase in the severity, incidence, and mortality rate of CAD, as evidenced by previous studies [40, 41]. A higher rate of hypertension, DM, dyslipidemia, and obesity has been found in older women in comparison to younger ones [42, 43]. Additionally, the prevalence of multiple risk factors significantly increases with aging [44]. Notably, in a study conducted in Tehran City, the younger patients who were diagnosed with CAD had more risk factors [45]. As a result, the CAD subjects had a greater number of risk factors than their age‐matched non‐CAD counterparts. Our findings also demonstrated an overall increasing trend in the number of risk factors with age. Further, the CAD patients had more risk factors than non‐CAD cases in the same age range.

The impact of non‐modifiable risk factors is consistent across males and females, in various geographic locations, and among different ethnicities. In Iran in 2005, 80,000 deaths were attributed to hypertension, 34,000 to hyperlipidemia, 34,000 to DM, and 11,000 to smoking in 2005 [46]. Moreover, a national study carried out in Iran in 2021 reported the prevalence rate of CAD risk factors using the STEPwise approach to non‐communicable disease risk factor surveillance (STEPS) [27]. The results of this study were as follows: 51.3% of the population had low physical activity, 19.4% were smokers, 32% had hypertension, 63% had a BMI ≥ 25, and 17.5% had DM. Systolic BP is the most critical modifiable risk factor contributing to some further CAD risks that occur with age [41]. According to a systematic review and pooled analysis published in 2016, Iranian women aged 25 and older had a prevalence of hypertension of 29% on a national scale [47]. Furthermore, TeCS recently reported a hypertension prevalence of 39.9% among adult women in Tehran [48]. Moreover, prior investigation results illustrated a higher rate of prevalence and incidence of hypertension among women over 60 [49]. In the present study, hypertension had a significant association with CAD. Overall hypertension prevalence was 29.5% in our study, which was in line with the general female population. It is noteworthy that females in our study were older than 35 years, which may influence the overall prevalence of hypertension. Additionally, the CAD‐positive group had a significantly higher prevalence of hypertension in comparison with the non‐CAD group (77.1% vs. 27.3%). In another study conducted in Tehran City on patients with obstructive CAD, the prevalence of hypertension among women was 76.2%, which was similar to our result [45]. Notably, compared to men, women are less likely to receive medication to treat hypertension and have poor BP control [50]. Additionally, women who receive antihypertensive treatments suffer from higher systolic BP than men. These findings support the fact that women are likely to be more prone to developing CAD from hypertension, highlighting the unmet need for further evaluations and community awareness.

Hyperlipidemia was the most prevalent risk factor in our study, with an overall prevalence of 34.6%, which was consistent with previous findings [27, 31, 32]. WHO reports indicate that dyslipidemia is a contributing factor to half of all ischemic heart disorders [51]. Further, a case–control study conducted in 52 countries revealed that dyslipidemia, with a rate of 47.1%, had the highest population‐adjusted risk factor for atherosclerotic CVD among women [34]. In a study carried out in Yazd City, the overall dyslipidemia prevalence among the women’s population was 58.6%, which was higher than our result [52]. The prevalence rate of dyslipidemia in our study was significantly higher in CAD‐positive cases compared to non‐CAD peers (74.1% vs. 32.9%), which was in line with prior investigations confirming the potential of dyslipidemia in the development of CAD [31]. Moreover, our findings were similar to another study conducted in Tehran City in which the overall prevalence of dyslipidemia among CAD‐positive women was 75.3%. It is imperative to note that dyslipidemia is common in the female gender [38]. Moreover, hormonal changes after the menopause period can increase the risk of dyslipidemia in women. Therefore, improving the screening and treatment of dyslipidemia in women becomes mandatory.

Our study’s overall DM prevalence rate was 18% with a significant difference between CAD and non‐CAD cases (47.8% vs. 14.4%). Despite the relatively low prevalence of DM in Asia, Middle Eastern countries such as Iran and Saudi Arabia are also regarded as major hotspots of the global DM epidemic [20]. According to WHO data in 2020, the overall prevalence of DM in Iran was 10.3%, with men and women exhibiting a prevalence of 9.6% and 11.1%, respectively [53]. Moreover, previous studies have demonstrated an increase in the prevalence of DM in adults between 25 and 65 years, as well as a comparison to the worldwide average [54]. Prior investigations in Yazd City have revealed a DM prevalence of 20.5% and 15.4% among the female population [27, 55]. Moreover, DM prevalence has been reported in 10.3% of women aged 15–75 years in Kerman City [55]. This difference in the prevalence of DM indicates that multiple factors, such as genetic predisposition, environmental factors, and psychosocial status, influence DM incidence. In women, DM diminishes the protective effects of estrogen against CAD; therefore, diabetic women experience a 4.3‐fold higher CAD incidence and mortality in comparison with a 2.7‐fold higher risk in men [34]. Moreover, diabetic women in the premenopausal period experience the same risk of CAD development as their male counterparts [43]. Considering these findings, it is clear that DM is a significant public health challenge, particularly in the female population, necessitating a comprehensive approach to timely diagnosis and management.

In our study, alcohol and tobacco use, BMI, physical activity, and educational level were not significantly associated with CAD. These findings were inconsistent with previous studies reporting obesity, central obesity, smoking, sedentary behavior, and lower educational attainment as important risk factors for CAD development [9, 27, 43, 52, 56]. Nonetheless, in our survey, a higher proportion of CAD subjects reported smoking, a higher waist‐to‐hip ratio, lower physical activity, and a lower educational level, findings consistent with other studies [9, 57]. With respect to our results, important socioeconomic and educational disparities were also observed in the baseline analyses in our study, where women with CAD were more frequently illiterate, had lower educational attainment, and were more commonly housewives or retired compared with non‐CAD participants. Consistently, previous studies have demonstrated that lower educational attainment and socioeconomic disadvantage are associated with poorer cardiovascular outcomes, reduced health literacy, delayed healthcare utilization, lower adherence to preventive strategies, and a greater burden of cardiometabolic risk factors [58–60]. The lack of independent associations for several lifestyle‐related variables in the adjusted model may reflect the complex interplay between socioeconomic determinants, aging, and established cardiometabolic comorbidities, particularly diabetes, hypertension, and hyperlipidemia, which remained strongly associated with CAD in our study. Furthermore, given the cross‐sectional design of the present study, causal relationships between socioeconomic factors and CAD could not be fully established. Additionally, the age distribution pattern and single‐gender base of this study may explain this discrepancy. Lastly, future longitudinal analyses during the follow‐up phases of the TeCS may provide a more comprehensive understanding of the long‐term impact of socioeconomic and educational determinants on CAD development and progression among women in Tehran.

### 4.1. Strengths and Limitations

This study has several notable strengths. This study is the first to investigate the epidemiology of CAD and its associated risk factors in a large, representative sample of women from all geographical regions of Tehran. Additionally, by comparing risk factors between CAD and non‐CAD participants, the study enhances the accuracy of reported associations.

However, several limitations should be acknowledged. As a cross‐sectional study, risk factors were assessed at a single baseline visit, and non‐responders may have influenced the reported prevalence. Although TeCS used a multi‐stage sampling design, our current analyses were unweighted and did not adjust for sampling variance via Taylor‐series linearization, as the primary aim was to evaluate associations rather than generate population‐level estimates. Future analyses will incorporate design‐based adjustments to improve external validity. Listwise deletion was applied due to low missingness (< 5%) and the absence of a discernible bias pattern, but multiple imputation will be considered in subsequent analyses. Other limitations include the lack of precise data on the age of CAD onset and disease duration, restricting the ability to assess temporal progression. Data on treatment patterns, medication use, postprandial glucose levels, menopausal status, age at menopause onset, details of menopausal surgeries (e.g., oophorectomy, hysterectomy), and hormone replacement therapy were also unavailable, limiting the depth of analysis. Despite these limitations, the observed prevalence of CAD among adult women in Tehran is concerning. Our findings align with prior studies demonstrating the influence of both modifiable and non‐modifiable risk factors on CAD risk, with certain factors showing particularly strong associations in prediabetic individuals. Future follow‐up studies will address these gaps, enabling a more comprehensive understanding of CAD epidemiology and determinants in this population.

## 5. Conclusion

Our study found a CAD prevalence of 4.48% among women in Tehran, with an age‐weighted prevalence of 4.1%. Older age, DM, hypertension, and hyperlipidemia were identified as independent determinants of CAD in this population. Given that CAD is a leading cause of death among Iranian women, these findings can inform healthcare policymakers in developing strategies for earlier diagnosis, targeted treatment, and prevention. In particular, interventions should focus on high‐risk women with multiple comorbidities to reduce the overall burden of CAD.

## Funding

This study was financially supported by the Iranian Ministry of Health and the Tehran Heart Center.

## Ethics Statement

This project was approved by the Tehran Heart Center’s review board and the ethical committee of the Tehran University of Medical Sciences (ID: IR.TUMS.MEDICINE.REC.1399.074). All procedures followed were in accordance with the ethical standards of the responsible committee on human experimentation (institutional and national) and with the Helsinki Declaration of 1975, as revised in 2000. Informed consent was obtained from all study participants before enrollment.

## Consent

Please see Ethics Statement.

## Conflicts of Interest

The authors declare no conflicts of interest.

## Data Availability

All the data generated or analyzed during the current study are available from the corresponding author upon reasonable request.
